# The Relationship between Job Insecurity and Psychological Well-Being among Malaysian Precarious Workers: Work–Life Balance as a Mediator

**DOI:** 10.3390/ijerph20032758

**Published:** 2023-02-03

**Authors:** Nurul Iman Abdul Jalil, Soon Aun Tan, Nur Shakila Ibharim, Anisah Zainab Musa, Siew Hui Ang, Wustari L. Mangundjaya

**Affiliations:** 1 Department of Psychology and Counselling, Faculty of Arts and Social Science, Universiti Tunku Abdul Rahman, Kampar 31900, Malaysia; 2 Faculty of Psychology, Universitas Bhayangkara Jakarta Raya, Jakarta 12500, Indonesia

**Keywords:** precarious worker, psychological well-being, job insecurity, work-life balance

## Abstract

The emergence of coronavirus disease has impacted human lives, one of which is economic disruption. Many Malaysian organisations have devised various crisis-response techniques, such as downsizing, laying off, retrenching, and combining. As a result, the number of Malaysians working in precarious jobs, which are defined by unpredictable and uncertain situations, has indirectly increased, hence increasing job insecurity. Therefore, maintaining psychological health is essential to safeguarding the mental health of employees. In the current working landscape, job security and work–life balance have commonly been deemed necessary in contributing to well-being among employees. As a result, the purpose of this study was to examine how work–life balance influences the relationship between job insecurity and psychological well-being among Malaysian precarious workers. It also fills a gap in the research by explaining the causal association between job insecurity and psychological well-being among precarious workers, as previous well-being studies have largely focused on employees with secure jobs. There were 442 responders collected using purposive and snowball sampling methods, and they were requested to complete the Job Insecurity Scale (JIS), Work–Life Balance, and Well-Being Index Scale (WHO-5). Job instability was negatively connected with work–life balance and psychological well-being. On the other hand, work–life balance was found to be positively related to psychological well-being. This supports the notion that work–life balance is a significant mediator in the relationship between job insecurity and psychological well-being. These findings emphasise that Malaysian precarious workers with job security could enhance their psychological well-being by achieving work–life balance.

## 1. Introduction

### Study Background

The emergence of COVID-19 has had a massive impact on human life, one of which is economic status. Many countries implemented lockdowns and social distancing measures to counteract the contagion, with Malaysia employing a different approach—a movement control order (MCO) [1]. While these policies were deemed helpful in combating the pandemic, they indirectly forced the closure of many businesses, resulting in an economic crisis. As a result, organisations’ daily operations stopped, affecting revenue and income. It was found that over 37,000 small to medium-sized and more than 200 sport-related companies closed their business, claiming that they would require financial assistance to continue their daily operations [2]. Therefore, large-scale layoffs were implemented to cope with the financial crisis experienced by organisations, with the International Labour Organization (ILO) claiming that 94% of the worldwide workforce was affected by forced or advised workplace closures in September 2020 [3]. In Malaysia, more than 15,000 employees lost their employment in line construction and office work [4], while in Sarawak, about 4000 people have been unemployed since MCO was implemented [5]. The employment rate has also been affected by other methods, such as merging and restructuring [6].

Maintaining one’s work through economic downturns is complex, and when unemployment is rampant, getting work becomes even more challenging. One alternative way workers seek to sustain their lives during the pandemic is to engage in precarious employment, making the unconventional and gig labour market more prevalent [7]. Precarious work refers to any nonstandard work, such as part-time, contract-based, freelance, self-employment, or dispatched, all of which are characterized by unpredictability, instability, and insecurity. In Malaysia, 28,600 locals are engaging in various precarious jobs, such as e-hailing and food delivery, during the pandemic, lowering their quality of life and having unfixed salaries [8]. They are often associated with low earnings that can influence housing demands, lack of social and health insurance, bargaining power, rights such as social security, and exposure to unfavourable working circumstances [9].

Previous research on precarious workers in Malaysia has focused on migrants such as Rohingya refugees, Nepali, Pakistani, Bangladeshi, Indonesian, and Burmese [10,11,12,13,14,15]. This group of precarious workers has raised researchers’ interest because they experience lots of employment issues and challenges as well as their lack of protection under labour law [3]. This would not only affect worker’s ability to do their jobs but would also have an impact on their health and well-being, such as being in stressful situations or causing them to have poor physical health [16]. However, when it comes to precarious worker issues among Malaysian citizens, limited studies have been done. One by Nor [17] was conducted to explore the precarious employment experience among low-income single mothers in Malaysia. Other studies by Asgali and Abd Hamid [18] have been done, but focused on the role of the government in precarious work among fishermen in Semporna, Sabah.

Precarious employment has been identified as a job stressor for workers, with research indicating unfavourable health outcomes [19,20]. For instance, Italian workers on temporary contracts were found to have a higher risk of acquiring mental disease [21], demonstrating that precarious workers eventually experience psychological issues that have an impact on their psychological well-being (PWB). PWB is described as the experience of pleasant affect being greater than the experience of negative emotion, with the absence of mental illness, and can improve life functioning [22]. Given the possibility of poor mental health among precarious workers prior to the pandemic [21], this scenario is predicted to worsen in the presence of infection. This is because all workers are susceptible to stressful economic risks and fluctuations in the labour market, such as the high risk of unemployment in Malaysia [4]. These challenges then increase job insecurity, which refers to the possibility of losing one’s job or the stability of one’s employment [23]. Precarious workers are especially vulnerable since they are typically associated with short-term employment and variable working hours. Throughout the epidemic, some instances, such as contracts not being renewed and being forced to reduce working hours, have been noticed among contract-based workers, which may heighten feelings of unemployment without being formally laid off [7]. As a result, the heightened level of job insecurity causes employees to be unclear about their future, whether they will lose their jobs or continue to work, and unfortunately, they lack the power and resources to control this [24]. This could eventually affect their mental health, leading to poor PWB [25,26]. Previous studies have frequently explored the impacts of job instability on PWB [26,27,28], with the intended population being employees with stable employment. Previous academics have requested further research into the well-being of precarious workers, which has gone unnoticed [29]. Sutarto [30] also stated that ergonomic circumstances have been underappreciated in regard to psychosocial issues such as job instability. As a result, emphasizing the link between work insecurity and PWB is critical so that organisations can pay more attention to their employees’ job insecurity.

In vocational psychology, it is unclear how job instability is related to PWB, with Lee and Tai [31] recommending mediation research to better understand the relationship by incorporating work–life balancing practices. While prior research revealed a correlation between job instability and PWB, Yang et al. [32] discovered that work–life balance mediates the relationship. Previous research has employed work–life balance as a moderator in assessing organisational outcomes [33,34]. In this respect, many employees are still far from achieving it in the current working environment in Malaysia. Likewise, precarious employees have also struggled with work–life balance because one potential strategy for workers to reduce job insecurity is increasing their working hours to indicate their efforts to protect their employment [35]. Long working hours, on the other hand, may make it difficult for employees to combine work and life commitments [36], making work–life balance harder to attain [37]. As indicated by the literature and in the current study, work–life balance is often highlighted among working mothers and underlined in research tied to the family rather than organisational lines [38,39]. As a result, the purpose of this study was to fill gaps by studying the mediating effects of work–life balance, as proposed by Lee and Tai [31]. This would educate non-governmental organisations, human resource managers, and organisations about how work–life balance can harm one’s psychological well-being during a pandemic.

The association between job insecurity, work–life balance, and PWB can be explained through the conservation of resources (COR) theory. This denotes that stress is an imbalanced state between environmental demand and the capacity of an individual to respond to it. The primary rules of COR theory indicate that people always try to preserve, acquire, boost, and protect any valuable resource. There are two fundamental principles: the primacy of loss and resource investment. The primacy of loss relates to individuals’ protection of resources against loss, showing that resource loss is more important than resource gain. In contrast, the resource investment principle indicates that individuals invest more resources to preserve and recover from resource loss, as well as obtain resources. Within this development, COR postulates that individuals experience psychological stress when (i) resources are lost, (ii) resources are threatened, or (iii) resources fail to appreciate after investment [40].

In the present study, job insecurity can be identified as a significant job stressor negatively associated with employees’ mental health outcomes [36], in which precarious employment is placed with threatened loss. Nevertheless, job security is a valuable resource because it is crucial for attaining other basic needs and social status [41]. When job insecurity arises, income stability may also be affected [42]. In accordance with the COR theory, when a resource (e.g., employment) is threatened with loss, it exacerbates stress as an emotional response to deal with it. Such negative emotion can be overwhelming, thus negatively affecting well-being [43].

The resource investment principle of COR theory claims that individuals strive to invest more resources to protect against resource loss, and stress arises when resources fail to appreciate after investment [40]. This study argues that precarious workers may invest or take up additional resources, such as time and energy in their current employment, to protect against potential resource loss, that is, job insecurity, and that causes stress [35].

## 2. Literature Review

### 2.1. Psychological Well-Being

Based on the eudaemonic assumption, PWB is defined as one’s inter- and intra-functioning in a positive way, such as the sense of connectedness with others and self-reflexive attitudes like the sense of mastery and personal development [44]. It also has to do with improving one’s mental health, which results from healthy functioning at its best [45]. To further understand PWB, it can be derived from the concept of the mature personality, Rogers’s fully functioning individual, and Maslow’s notion of self-actualisation. There is autonomy, personal growth, self-acceptance, purpose in life, environmental mastery, and positive relatedness with others. Maslow’s theory describes self-actualisation and fully functioning individuals as requiring self-acceptance. It also emphasises the ability to establish valuable connections with people and high emotional intelligence skills. Environmental mastery denotes the ability to function under pressure, whereas life purpose denotes the ability to choose goals and generate a feeling of life direction, all of which contribute to one’s life’s meaning. Personal development refers to one’s capacity to expand and realise one’s inner potential. These PWB concepts, according to Ryff’s theory, are all crucial to an individual’s ability to realise and maintain a fully functional self [46].

Past studies emphasise a wide range of personal health and organisation outcomes resulting from high PWB, such as being more likely to stay active in physical activity [47] and decreasing the chance of having psychopathology [48]. In terms of work domains, higher PWB has been linked to more co-worker support among working people and acts as emotional support to gig workers’ functioning [49,50]. Furthermore, when working in a hazardous profession, drivers with higher PWB felt less anxious and agitated because they could concentrate on the good aspects of their everyday functioning, such as enjoying the scenery while driving. As a result, prior research reveals that PWB significantly improves mental health. To raise the degree of PWB among precarious workers in Malaysia, the associated factors of PWB and its potential direct influence as a mediator must be researched.

### 2.2. Job Insecurity and PWB

From the cognitive perspective, job insecurity (JI) refers to the threat to job employment, whereby JI is defined as the emotional response, such as worrying or being stressed, to the perceived threat of the job, such as in the affective perspective [51]. Job insecurity can also be classified as qualitative or quantitative. Quantitative JI makes assumptions about both the cognitive and emotive parts of JI, including the perception of uncertain employment and the anxiety brought on by the threat [52]. The perception of unfavourable job connection quality changes, such as a lack of career advancement or income increases, is defined as qualitative JI [42] (p. 182). It is a self-defined experience that can vary across people, a problem with the future, and an involuntary threat to one’s job. In the current study, work insecurity is defined as a perceived danger to the continuity and stability of employment from the perspective of quantitative job insecurity [53]. Furthermore, it occurs from insecure to secure, with employees experiencing job security when they believe the continuity and stability of their position are unaffected.

Job insecurity is strongly related to precarious work in this era and has been considered a stressor that can potentially increase work demands on employees [24]. Given that COVID-19 caused a global recession, the experience of job insecurity may have been exacerbated. Many workers were forced to reduce work hours, lost their jobs, and had to accept lower pay because of the organisation’s financial crisis, resulting in greater job insecurity in the current working landscape [54]. Such circumstances cause individuals to feel anxious, and that affects their life functioning. It has been found to negatively impact mental health and function among employees [27,28,55,56]. Precarious workers with high job instability reported psychological discomfort [26]. One potential explanation is that job insecurity makes individuals feel powerless and unable to control it [24]. They are unsure how to solve these changes, leading to poor mental well-being.

### 2.3. Work–Life Balance and PWB

Work and family are the two of the most important aspects of human life, yet they are two distinct realms with different norms, cognitive patterns, and actions, depending on employment qualities, as well as personal and family variables and conditions. Work and life are understood separately, with work referring to the effort and energy we expend to accomplish something and life referring to a combination of good and negative emotions, such as happiness. By balancing all of one’s responsibilities at work and home, work–life balance (WLB) is achieved [37]. That is, WLB is defined as a condition of equilibrium in which workers’ work demands and life duties are in terms of emotional, behavioural, and personal aspects. It is also based on the individuals’ subjective evaluation.

Even though the work–life balance is a simple concept, many working professionals struggle to recognise it. People spending more time at work is widespread in Asian countries since labour overtime has been characterized as a culture in Asian workplaces [57]. Such work–life imbalance is an unhealthy lifestyle because employees are overwhelmed with work demands, encountering conflict between work and life. A study by Mensah and Adjei [58] showed that European working adults with low work–life balance reported poorer physical and psychological health, with women being more affected than men. A similar result was found in the Asian teaching profession, with individuals who can handle work and life duties equally being more likely to cope with their emotions, resulting in greater PWB [59]. From the COR perspective, these findings can be explained by employees spending longer time at work as one resource to cope with their work responsibilities, thus negatively affecting their family role, such as less time being given to children, and eventually heightening their stress [35].

Given the nature of the work of precarious workers, it would lead to distress and may affect their PWB [60]. Precarious workers reported feeling powerless because they believed this was the only job they could get. They must have social support from family and friends, as well as employers, co-workers, and customers, to protect their PWB [61]. However, when they are required to work overtime, they have less time for life responsibilities and social interactions. Malaysians adhere to collectivist culture, so they deem interpersonal relations with family necessary to their life. Hence, it is crucial to identify how significant work–life balance plays a role in Malaysian precarious workers’ PWB.

### 2.4. Job Insecurity and Work–Life Balance

In today’s labour market, workplace uncertainty is often viewed as a substantial source of job stress [31]. When job insecurity arises, the worker’s working behaviour changes to work intensively to demonstrate their effort in the occupation [62]. According to the COR theory, when a person is stressed for a lengthy period, the rate of resource consumption increases [63]. Further resource loss may occur if these resources are not rapidly replenished [64]. In this vein, such work behaviour may have an impact on an individual’s life: Minnotte and Yucel [36] and Soelton et al. [28] discovered that US workers and Indonesia workers with significant job insecurity were more likely to struggle with balancing work and family responsibilities, which may have an impact on their health over time. Furthermore, higher working hours and work expectations were discovered to have a negative impact on workers’ work–life balance [28,62,65]. This indicates that when job insecurity occurs, further resources, that is, family and life demands, are affected.

Job insecurity is projected to be more prevalent among precarious workers due to their variable working conditions and nature. For example, comparitive research by Nielsen et al. [66] found that part-time workers faced more job insecurity than full-time workers due to a lack of control, weak rights, and terms. Moreover, casual workers were more inclined than permanent workers to commit to extended working hours [65]. These studies show that precarious workers are highly vulnerable to job insecurity. However, given that work–life balance is constantly regarded as a valuable resource by the current Malaysian workforce [67]. it is unclear to what extent job insecurity association with work–life balance exists among Malaysian precarious employment.

### 2.5. The Mediating Role of Work–Life Balance between the Association

Speculating from the COR theory, when employees are experiencing job uncertainty during the COVID-19 pandemic, they will invest more resources to cope with their job demands and insecurities. Consequently, workers’ resources will be drained if this situation persists, leading to poor mental well-being [31]. When people experience employment uncertainty, they also face the possibility of losing their ability to support themselves and their families. Workers may expend greater time and energy in their job to safeguard their employment in order to protect their fundamental food and income needs [35]. Tentatively, spending more time at work reduces the available time for family, making it hard to achieve work–life balance, and thus having limited time to engage in social interaction [62,68]. Research has also shown an association between job insecurity, job demands, and work–life balance [62,69,70].

Furthermore, during the pandemic, employees may face additional challenges like setting aside additional time for domestic tasks and childcare and dealing with the new working practice [30]. These difficulties make it harder to achieve a work–life balance [71]. As a result, past scholars proved that individuals with poor work–life balance reported weaker mental health [58,72], and poorer life adjustments such as sleep, attention, learning and remembering [73]. These studies have shown the association between job insecurity, work–life balance, and PWB. Past researchers have commonly taken work–life balance as a mediator in assessing organisational outcomes [33,34]. A recent study has found a mediating effect of work–life balance in workaholism and depression among workers in Hong Kong [32]. Therefore, this study examines the mediating role of work–life balance in the association between job insecurity and PWB among Malaysian precarious workers. This is critical to protect workers’ well-being because precarious workers with job insecurity experience poorer work–life balance than full-time employees [74].

### 2.6. The Present Study

**Figure 1:**
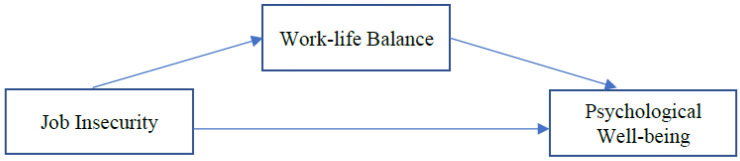
Focus of Current Study.

Figure 1 presents focus of current study. This study focused on job insecurity contributing to precarious workers’ PWB. According to the COR theory, individuals experience psychological stress when (i) resources are lost, (ii) resources are threatened with loss, or (iii) resources fail to appreciate after investment [40]. When job insecurity occurs, it acts as a stressor that influences ones’ employment with threatened loss, and so does the effect on income [42]. When a person is stressed for a lengthy period, the rate of resource consumption increases [63]. Further resource loss may occur if these resources are not rapidly replenished [64]. Therefore, when job insecurity arises, he or she would work more extended hours. After investing more time to work and resulting in less time for family demands, precarious workers fail to regain the resource of time and energy equally, thus causing work–life imbalance. Consequently, individuals’ stress heightens, and they may feel less socially connected, leading to poorer mental health [75].

In this study, job insecurity highlighted the overall perception of job insecurity during the last three months, work–life balance on reflecting over the precarious workers work and non-work activities over the past three months and psychological well-being by measuring the subjective psychological well-being of precarious Malaysian employees, such as cheerfulness.

### 2.7. Hypotheses

Job insecurity is negatively associated with psychological well-being among Malaysian precarious workers.

Work–life balance is positively associated with psychological well-being among Malaysian precarious workers.

Job insecurity is negatively associated with work–life balance among Malaysian precarious workers.

Work–life balance mediates the association between job insecurity and psychological well-being among Malaysian precarious workers.

## 3. Method

### 3.1. Participants

This study involved 442 Malaysian precarious workers aged between 19 and 64 (mean = 29.93; standard deviation = 8.84). The majority of the respondents were women (66.4%). The respondents comprised 55.9% Chinese, 36.3% Malay, 5.9% Indian and 1.9% other ethnic minorities, such as Bumiputera Sabah and Sarawak. More than half the respondents were single (51.2%), 41.7% were currently married, 5.9% were in a relationship, 0.9% were divorced, and 0.2% were widowed. The respondents consisted of 42.7% contract-based employment, part-time employment (32.2%), self-employment (14%), dispatched workers (9.2%) and agent workers (1.7%). The majority of the respondents reported household income below 40% (B40, 79.6%), followed by middle 40% (M40, 17.8%) and upper 20% (T20; 2.6%).

### 3.2. Procedure

The data of the study were collected using purposive and snowball sampling methods. An online survey was designed using Qualtrics, an online survey platform. A participant recruitment poster was created with the participation link and QR code. The poster was then posted on various social media platforms such as Facebook, LinkedIn, Instagram, etc. Also, potential participants were approached individually by the research team and requested to circulate (i.e., snowballing) the research information and survey link to their networking that fulfils the research requirement. Participants were required to fulfil the criteria of a Malaysian who is currently a precarious worker. The definition of precarious work was made available in the survey post and in the survey question to ensure participants understood the concept of precarious work and were qualified to be the subject of the research interest of the present study. Research information was accessible on the first page of the survey link, followed by the informed consent form. Each respondent who completed the survey was compensated with a RM 5 (Ringgit Malaysia) Touch’ N Go E-reload pin. The ethical clearance for the study procedure was reviewed and approved by the Institutional Scientific and Ethical Review Committee.

### 3.3. Measures

Job insecurity was assessed using the job insecurity scale developed by Jung et al. [76]. This eight-item scale is rated from 1 (strongly disagree) to 7 (strongly agree). A mean score was computed after reverse scoring for questions 1 to 4. A higher mean score indicated higher job insecurity. The Cronbach alpha reliability was reported at 0.64.

The work–life balance scale [77] consists of four items used to measure work–life balance. Respondents were requested to rate on a 5-point Likert scale from 1 (strongly disagree) to 5 (strongly agree). Question 2 is the reserve item. A mean score was computed after reverse scoring for question 2. A higher mean score corresponded to a higher work–life balance. Cronbach alpha reliability was 0.65.

PWB was measured using the WHO-5 well-being index scale (WHO-5) [78]. This scale consists of a five-item rating from 0 (at no time) to 5 (all of the time). Respondents were asked to indicate their psychological state over the last two weeks. A mean score was computed with a higher score indicating higher PWB. Cronbach alpha reliability was 0.74.

### 3.4. Data Processing and Analysis Plan

IBM SPSS was used to run the analysis of the study. The data were first examined for the associations between variables of the study using Pearson correlation analysis. Hayes’s (2013) PROCESS Marco for SPSS, version 4.0 with Model 4 was then used to examine the hypothetical mediating role of work–life balance in the association between job insecurity and PWB among precarious worker respondents. Age and gender were set as covariates in the present analysis. A 95% bias-corrected confidence interval (CI) was examined using 10,000 bootstrap samples. The indirect effect was significant if both CIs were in the same direction.

## 4. Results

### 4.1. Correlation between Variables of the Study

Table 1 presents the descriptive statistics and correction results among the variables. The mean score for job insecurity was 4.03 (SD = 0.87), work–life balance was 2.96 (SD = 0.77), and psychological well-being was 3.16 (SD = 0.87). The results of correlation revealed that job insecurity was negatively associated with work–life balance and PWB. Inversely, work–life balance was positively linked to PWB. Specifically, precarious worker participants who reported higher job insecurity tend to have a lower work–life balance and PWB. A moderate and significant positive link was also found between work–life balance and PWB.

### 4.2. Mediation Role of Work–Life Balance

**Figure 2:**
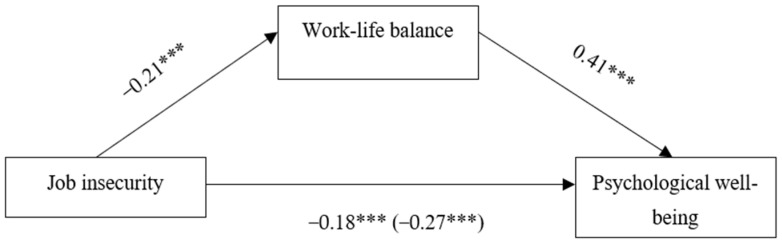
Mediation model showing the effect of job insecurity and work–life balance on psychological well-being. The values shown are unstandardised coefficients. The total effect is shown in the parentheses. *** *p* < 0.001.

Figure 2 presents the results of the mediation analysis. Job insecurity was found to be negatively linked to work–life balance, *B* = −0.21, *SE* = 0.04, *t*(418) = −4.91, *p* < 0.001, 95% *CI* [−0.29, −0.12]. In the opposite direction, work–life balance was positively linked to PWB, *B* = 0.41, *SE* = 0.05, *t*(417) = 8.07, *p* < 0.001, 95% *CI* [.31, 0.51]. The result of the direct effect of job insecurity was negatively significant even after controlling the effects of work–life balance together with the two covariances (i.e., age and gender), *B* = 0.18, *SE* = 0.05, *t*(417) = −4.13, *p* < 0.001, 95% *CI* [−0.27, −0.10]. In the total effect model, job insecurity was negatively associated with PWB, unstandardised regression coefficient, *B* = −0.27, *SE* = 0.05, *t*(418) = [−5.76, *p* < 0.001, 95% *CI* [−0.36, −0.18]. The mediating role of work–life balance on the link between job insecurity and PWB was significant, *B* = −0.08, *SE* = 0.02, 95% *CI* [−0.13, −0.04], indicating work–life balance as a significant mediator.

## 5. Discussion

The current study aimed to examine the mediating role of work–life balance in the association between job insecurity and PWB among Malaysian precarious worker respondents. The findings supported the notion that job insecurity was negatively associated with work–life balance and PWB, whereby work–life balance was positively linked to PWB, supporting H1, H2, and H3. Furthermore, the finding also indicated that work–life balance mediates the relationship between job insecurity and PWB, supporting H4.

Firstly, Malaysian precarious workers who experienced high job insecurity were more inclined to poorer work–life balance and lower PWB, which are in line with the literature [28,36]. According to the COR theory, given that job insecurity is a significant stressor to employees [31], Malaysian precarious workers could say that they work harder to secure their current employment by showering more effort, thereby reducing the level of stress. However, when more time is taken into the current employment, less time is paid to the family. Therefore, it influences family and work demands [62], making their work and life imbalanced. Highly job-insecure precarious workers are at risk of experiencing weak PWB [55,56]. This could be explained by the sense of powerlessness [24], in which Malaysian precarious workers have no idea how to control the negative circumstance and feel powerless to make any changes to preserve their employment. As a result, being surrounded by such negative emotions may drain workers’ mental health, thereby leading to poorer life functioning [26]. Furthermore, these findings are linked to the feature of precarious labour employment. Since insecure work is frequently irregular and unpredictable [8], they may have poor salaries, affecting their living expenses. As a result, individuals must commit to additional work in order to secure their employment and, at the very least, have some money to cover their basic needs. Precarious workers, unlike permanent workers, typically lack social and health insurance [9], which may prevent them from obtaining counselling or medical aid while experiencing high-stress levels due to employment insecurity. As a result, they may eventually neglect their mental health rather than invest money to seek professional care.

Secondly, Malaysian precarious workers with poor work–life balance are also associated with poor PWB, or vice versa. In adhering to the Malaysian collectivist society that views interpersonal relations as necessary to their life, workers with work–life imbalance are usually overwhelmed with their work demands, and thus have less time as an available resource to invest in building social interactions and connectedness with their families and friends [35]. Such a lack of connectedness may further heighten workers’ stress and eventually affect their PWB [61]. Conversely, if workers can achieve a work–life balance, they have broader resources to reach out to, thereby managing their emotions more effectively, contributing to their better PWB [59].

The results of the study also supported work–life balance being a significant mediator that explains the indirect relationship between job insecurity and PWB among Malaysian precarious workers. This result implies that precarious workers with higher job insecurity tend to report a lower work–life balance, resulting in lower PWB, based on the COR theory [40]. Malaysian precarious workers who experience job insecurity face not only their employment being threatened but other basic needs such as food and income being lost [42], while to preserve them from losing, precarious workers may invest other resources such as longer working time and effort in their precarious employment [62]. Conversely, the additional time and effort added to work is directly proportionate to the worker having less time and resources to spare for family demands and making good social relationships, thereby putting work–life balance at risk [36]. In the COR theory, individuals experience psychological stress when resources fail to appreciate after investment [40]. In this regard, the long working time and effort added to support their work demand may be drained and fail to restore, eventually heightening stress as an emotional response, reducing their PWB [31]. As a result, even though precarious employment is characterised by freedom and flexible working hours and arrangements, it is unlikely to be regarded as an added value to those who face high job insecurity during the pandemic because work–life imbalance appears to be a common strategy for them to reduce job insecurity.

## 6. Limitations and Recommendations for Future Studies

Some limitations need to be highlighted. Firstly, each measurement’s data were self-reported by the participants, which might favour social desirability biases [79]. This could potentially reduce the validity of the result. Therefore, it is suggested to incorporate alternative sources of data. For example, to understand the work–life balances among precarious workers, future research may conduct in-depth interviews with the respondents or their family members to understand their working hours and working and non-working life [80]. More sources of data collection may help in gathering more insightful information that can enhance validity of the research. Furthermore, given that Malaysians still adhere to traditional gender roles in which men are the primary breadwinners and that males were more distressed than mothers during the pandemic [81], this demonstrates the possible effect of gender on workers’ PWB. Gómez-Baya et al. [82] also found a substantial gender difference in attending to basic need fulfilment. Future research may add gender as a control variable to reduce confounding variables and obtain accurate results on how work–life balance is viewed as a mediator in impacting the association between job insecurity and PWB.

## 7. Implications

The findings of this study supported the COR theory in interpreting the relationship between job insecurity and psychological well-being with the primacy of loss and how, based on the primacy of resource investment, work–life balance serves as a mediator. These can contribute to the literature focusing on psychological well-being in insecure job employments in conjunction with other potential social determinants. The findings of the study also can be used to reform the existing public policies in order to sustain the work–life balance and psychological well-being of precarious workers, as this is in line with one of the policies of the Ministry of Human Resources, Malaysia, to provide social security protection for the well-being of employees, families, communities and the country [83]. Given that limited studies have focused on Malaysian precarious workers, this study can serve as a preliminary study for future researchers, thus deepening understanding of Malaysian precarious workers.

Furthermore, the findings of this study are useful for Malaysian organizations, governments, and policymakers by providing insight into how job insecurity is related to psychological well-being, as well as the underlying mediator that generates the association. Organizations can gradually become more employee-centric by introducing relevant employee well-being interventions that aid in achieving work–life balance, such as family supportive supervisory behaviour (FSSB) training, which has been proven beneficial for employees in previous studies [84]. On the other hand, the government may become involve in policy-making concerning employment benefits such as health, insurance and pensions. In addition, the government can launch campaigns emphasising job security’s importance and how to improve work–life balance. Lastly, policymakers may incorporate more policies relating to work–life balance, such as family leave for precarious workers who receive less social protection regularly, rather than giving it as a benefit for permanent workers. It will eventually contribute to their psychological well-being.

## 8. Conclusions

This study aimed to examine the mediating role of work–life balance in the association between job insecurity and PWB among Malaysian precarious employees. The study’s findings have demonstrated how important work–life balance and job security are to ensuring the mental health and psychological well-being of precarious workers. The findings of this study also add to the literature by employing the conservation of resources as the primary theory for explaining the potential correlations. It delves deeper into how job insecurity affects PWB among Malaysian precarious employees and underlines the need to build work–life balance in today’s working environment. As a result, during the epidemic, the government, organizations, or policymakers may push additional interventions or policies related to work–life balance to improve precarious workers’ PWB. Future researchers, however, should explore incorporating other theories, such as the job-demand resource theory, and include moderators to explain the underlying relationship between the variables. It is also necessary to re-examine and pay attention to the concept of work–life balance among precarious employees by including various contexts, locations, and cultures.

## Figures and Tables

**Table 1:** Descriptive statistics and correlation for variables.

Variable	1	2	3	4	5
1. Age	1				
2. Gender (1 = Male)	0.12 **	1			
3. Job insecurity	−0.01	−0.06	1		
4. Work–life balance	0.01	−0.03	−0.23 ***	1	
5. Psychological well-being	−0.06	−0.08	−0.27 ***	0.41 ***	1
Mean	29.93	-	4.03	2.96	3.16
Standard Deviation	8.84	-	0.87	0.77	0.87
Cronbach Alpha	-	-	0.64	0.65	0.74
Range	19–64	-	1–7	1–5	0–5

Note. ** *p* < 0.01; *** *p* < 0.001.

## Data Availability

The datasets generated during and/or analysed in this study are available from the corresponding author on request.

## References

[1] Marzo R.R., Vinay V., Bahari R., Chauhan S., Ming D.A., Johnson C.C., Thivakaran A.Q., Rahman M.M., Goel S. (2021). Depression and anxiety in Malaysian population during third wave of the COVID-19 pandemic. Clin. Epidemiol. Glob. Health.

[2] Augustin S. Over 37,000 Businesses Shut Down during MCO 3.0. https://www.freemalaysiatoday.com/category/nation/2021/09/28/over-37000-businesses-shut-down-during-mco3-0/.

[3] International Labour Organization (2020). ILO Monitor: COVID-19 and the World of Work. 6th ed. Updated Estimates and Analysis. https://www.ilo.org/wcmsp5/groups/public/-dgreports/-dcomm/documents/briefingnote/wcms_755910.pdf.

[4] Kaur M. 156,000 Layoffs Since Start of Covid-19 Pandemic. https://www.freemalaysiatoday.com/category/nation/2021/09/23/156000-layoffs-since-start-of-covid-19-pandemic/.

[5] Jee N. 4000 Sarawakians Lost Jobs Amid Covid-19, Assembly Told. https://www.freemalaysiatoday.com/category/nation/2022/05/18/4000-sarawakians-lost-jobs-amid-covid-19-assembly-told/.

[6] Papandrea D., Azzi M. Managing Work-Related Psychosocial Risks During the COVID-19 Pandemic. https://www.ilo.org/wcmsp5/groups/public/---ed_protect/---protrav/---safework/documents/instructionalmaterial/wcms_748638.pdf.

[7] Matilla-Santander N., Ahonen E., Albin M., Baron S., Bolíbar M., Bosmans K., Burström B., Cuervo I., Davis L., Gunn V. (2021). COVID-19 and precarious employment: Consequences of the evolving crisis. Int. J. Health Serv..

[8] Azman N.H. Underemployment Remains a Concern amid Lowest Unemployment Rate. https://themalaysianreserve.com/2022/01/11/underemployment-remains-a-concern-amid-lowest-unemployment-rate/.

[9] Kreshpaj B., Orellana C., Burström B., Davis L., Hemmingsson T., Johansson G., Kjellberg K., Jonsson J., Wegman D.H., Bodin T. (2020). What is precarious employment? A systematic review of definitions and operationalizations from quantitative and qualitative studies. Scand. J. Work Environ. Health.

[10] Nungsari M., Flanders S., Chuah H.Y. (2020). Poverty and precarious employment: The case of Rohingya refugee construction workers in Peninsular Malaysia. Humanit. Soc. Sci. Commun..

[11] Wahab A., Hamidi M. (2022). COVID-19 pandemic and the changing views of mobility: The case of Nepal–Malaysia migration corridor. Comp. Migr. Stud..

[12] Sunam R. (2022). Infrastructures of migrant precarity: Unpacking precarity through the lived experiences of migrant workers in Malaysia. J. Ethn. Migr. Stud..

[13] Khoso A., Thambiah S., Hussin H. (2020). Social practices of Pakistani migrant workers in Malaysia: Conserving and transforming transnational affect. Emot. Space Soc..

[14] Puder J. (2019). Excluding migrant labor from the Malaysian bioeconomy: Working and living conditions of migrant workers in the palm oil sector in Sabah. Austrian J. South-East Asian Stud..

[15] Viajar V.D. Dimensions of Precarity of Migrant Domestic Workers: Constraints and Spaces in Labor Organizing in Malaysia. https://gluasiaalumninetwork.files.wordpress.com/2016/08/glu-asia-alumni-workshop_2016.pdf.

[16] Kalleberg A.L., Vallas S.P. (2018). Probing precarious work: Theory, research, and politics. Res. Sociol. Work.

[17] Nor Z.M. Precarious employment amongst low income single mothers in Malaysia: The implications on family well-being. Proceedings of the E3S Web of Conferences.

[18] Asgali A., Abd Hamid F. (2018). The role of the government on precarious work among fishermen in Semporna, Sabah. MANU J. Pus. Penataran Ilmu Dan Bhs. (PPIB).

[19] Bodin T., Caglayan C., Garde A.H. (2019). Precarious employment in occupational health—An omega-networking group position paper. Scand. J. Work Environ. Health.

[20] Rönnblad T., Grönholm E., Jonsson J., Koranyi I., Orellana C., Kreshpaj B., Chen L., Stockfelt L., Bodin T. (2019). Precarious employment and mental health: A systematic review and meta-analysis of longitudinal studies. Scand. J. Work Environ. Health.

[21] Moscone F., Tosetti E., Vittadini G. (2016). The impact of precarious employment on mental health: The case of Italy. Soc. Sci. Med..

[22] Zhang Q., Zhang Z., Sun M. The mental health condition of manufacturing front-line workers: The interrelationship of personal resources, professional tasks and mental health. Proceedings of the 13th Global Congress on Manufacturing and Management (GCMM 2016).

[23] McGuinness S., Wooden M., Hahn M.H. (2012). Job insecurity and future labour market outcomes. Ind. Relat. J..

[24] Witte H.D. (1999). Job insecurity and psychological well-being: Review of the literature and exploration of some unresolved issues. Eur. J. Work Organ. Psychol..

[25] Mohamed A.F., Isahak M., Awg Isa M.Z., Nordin R. (2022). The effectiveness of workplace health promotion program in reducing work-related depression, anxiety and stress among manufacturing workers in Malaysia: Mixed-model intervention. Int. Arch. Occup. Environ. Health.

[26] Russo C., Terraneo M. (2020). Mental well-being among workers: A cross-national analysis of job insecurity impact on the workforce. Soc. Indic. Res..

[27] Bentzen M., Kenttä G., Richter A., Lemyre P. (2020). Impact of job insecurity on psychological well- and ill-being among high performance coaches. Int. J. Environ. Res. Public Health.

[28] Soelton M., Amaelia P., Prasetyo H. Dealing with job Insecurity, work stress, and family conflict of employees. Proceedings of the 4th International Conference on Management, Economics and Business (ICMEB 2019).

[29] Hussein N., Ishak N.A., Hussain I.A. (2018). Precarious work behaviour among millennial generation in Malaysia: A preliminary investigation. Turk. Online J. Des. Art Commun..

[30] Sutarto A.P., Wijayanto T., Afiah I.N. (2022). Exploring the mediation role of employees’ well-being in the relationship between psychosocial factors and musculoskeletal pain during the COVID-19 pandemic. Work.

[31] Lee M.H., Tsai H.Y. (2022). A study of job insecurity and life satisfaction in COVID-19: The multilevel moderating effect of perceived control and work–life balance programs. J. Mens. Health.

[32] Yang X., Qiu D., Lau M.C., Lau J.T. (2020). The mediation role of work-life balance stress and chronic fatigue in the relationship between workaholism and depression among Chinese male workers in Hong Kong. J. Behav. Addict..

[33] Gaikwad S., Swaminathan L., George S. Impact of work-life balance on job performance-analysis of the mediating role of mental well-being and work engagement on women employees in IT sector. Proceedings of the 2021 International Conference on Decision Aid (DASA).

[34] Varias V., Seniati A.N.L. (2017). The role of work-life balance as a mediator between psychological climate and organizational commitment of lecturers in higher education institutions. Divers. Unity: Perspect. Psychol. Behav. Sci..

[35] Bosmag Z., Liu H., Yu H., Wu Y., Chang S., Wang L. (2017). Associations between occupational stress, burnout and well-being among manufacturing workers: Mediating roles of psychological capital and self-esteem. BMC Psychiatry.

[36] Minnotte K.L., Yucel D. (2018). Work–family conflict, job insecurity, and health outcomes among US workers. Soc. Indic. Res..

[37] Sirgy M.J., Lee D.J. (2018). Work-life balance: An integrative review. Appl. Res. Qual. Life.

[38] Jayasingam S., Lee S.T., Mohd Zain K.N. (2021). Demystifying the life domain in work-life balance: A Malaysian perspective. Curr. Psychol..

[39] Yu S. (2014). Work–life balance—work intensification and job insecurity as job stressors. Labour Ind. A J. Soc. Econ. Relat. Work.

[40] Hobfoll S.E. (1989). Conservation of resources: A new attempt at conceptualizing stress. Am. Psychol..

[41] Jahoda M. (1981). Work, employment, and unemployment: Values, theories, and approaches in social research. Am. Psychol..

[42] Hellgren J., Sverke M., Isaksson K. (1999). A two-dimensional approach to job insecurity: Consequences for employee attitudes and well-being. Eur. J. Work Organ. Psychol..

[43] Ishikawa Y., Kohara M., Nushimoto A. (2021). Job stress and mental health among social workers: Evidence from a field experiment at a public employment support institution in Japan. Jpn. Econ. Rev..

[44] Burns R. (2017). Psychosocial Well-being. Encyclopedia of Geropsychology.

[45] Michalos A.C. (2014). Encyclopedia of Quality of Life and Well-Being Research.

[46] van Dierendonck D., Díaz D., Rodríguez-Carvajal R., Blanco A., Moreno-Jiménez B. (2008). Ryff’s six-factor model of psychological well-being, a Spanish exploration. Soc. Indic. Res..

[47] Kim B.Y., Kim W.S. (2022). The relationship between passionate behavior, psychological well-being, and intention to continue exercise of Pilates class participants. J. Korean Appl. Sci. Technol..

[48] Weiss L.A., Westerhof G.J., Bohlmeijer E.T. (2016). Can we increase psychological well-being? The effects of interventions on psychological well-being: A meta-analysis of randomized controlled trials. PLoS ONE.

[49] Apouey B., Roulet A., Solal I., Stabile M. (2020). Gig workers during the COVID-19 crisis in France: Financial precarity and mental well-being. J Urban Health.

[50] Jackman P.C., Henderson H., Clay G., Coussens A.H. (2020). The relationship between psychological wellbeing, social support, and personality in an English police force. Int. J. Police Sci. Manag..

[51] Jiang L., Lavaysse L.M. (2018). Cognitive and affective job insecurity: A meta-analysis and a primary study. J. Manag..

[52] Witte H.D. (2005). Job insecurity: Review of the international literature on definitions, prevalence, antecedents, and consequences. SA J. Ind. Psychol..

[53] Shoss M.K. (2017). Job insecurity: An integrative review and agenda for future research. J. Manag..

[54] Wilson J.M., Lee J., Fitzgerald H.N., Oosterhoff B., Sevi B., Shook N.J. (2020). Job insecurity and financial concern during the COVID-19 pandemic are associated with worse mental health. J. Occup. Environ. Med..

[55] Lee C., Huang G.-H., Ashford S.J. (2018). Job insecurity and the changing workplace: Recent developments and the future trends in job insecurity research. Annu. Rev. Organ. Psychol. Organ. Behav.

[56] Llosa J.A., Menéndez-Espina S., Agulló-Tomás E., Rodríguez-Suárez J. (2018). Job insecurity and mental health: A meta-analytical review of the consequences of precarious work in clinical disorders. An. Psicol..

[57] Tsai M., Nitta M., Kim S., Wang W. (2016). Working overtime in East Asia: Convergence or divergence?. J. Contemp. Asia.

[58] Mensah A., Adjei N.K. (2020). Work-life balance and self-reported health among working adults in Europe: A gender and welfare state regime comparative analysis. BMC Public Health.

[59] Prasad S.S., Sreenivas M. (2020). Role of work life balance on psychological wellbeing of the teaching professionals among Bangalore Institutions. Int. J. Indian Psychol..

[60] Kalleberg A.L. (2009). Precarious work, insecure workers: Employment relations in transition. Am. Sociol. Rev..

[61] Bosmans K., Hardonk S., De Cuyper N., Vanroelen C. (2016). Explaining the relation between precarious employment and mental well-being. A qualitative study among temporary agency workers. Work.

[62] Begum A., Shafaghi M., Adeel A. (2022). Impact of job insecurity on work–life balance during COVID-19 in India. Vision J. Bus. Perspect..

[63] Hobfoll S.E. (2001). Social support and stress. Int. Encycl. Soc. Behav. Sci..

[64] Guicciardi M., Pazzona R. (2020). The rebooting in sports and physical activities after COVID-19 Italian lockdown: An exploratory study. Front. Psychol..

[65] Bohle P., Quinlan M., Kennedy D., Williamson A. (2004). Working hours, work-life conflict and health in precarious and" permanent" employment. Rev. De Saúde Pública.

[66] Nielsen H.B., Gregersen L.S., Bach E.S., Dyreborg J., Ilsøe A., Larsen T.P., Garde A.H. (2021). A comparison of work environment, job insecurity, and health between marginal part-time workers and full-time workers in Denmark using pooled register data. J. Occup. Health.

[67] Murad D. Malaysians Want Work-Life Balance, Purpose, and Growth in Their Careers. https://www.thestar.com.my/news/focus/2021/11/28/malaysians-want-work-life-balance-purpose-and-growth-in-their-careers.

[68] Warner E., Andrews F.J. (2019). “Surface acquaintances”: Parents’ experiences of social connectedness and social capital in Australian high-rise developments. Health Place.

[69] Navajas-Romero V., Ariza-Montes A., Hernández-Perlines F. (2020). Analyzing the job demands-control-support model in work-life balance: A study among nurses in the European context. Int. J. Environ. Res. Public Health.

[70] Hussain A.B., Endut N. (2018). Do decent working conditions contribute to work–life balance: A study of small enterprises in Bangladesh. Asia Pac. J. Innov. Entrep..

[71] Carnevale J.B., Hatak I. (2020). Employee adjustment and well-being in the era of COVID-19: Implications for human resource management. J. Bus. Res..

[72] Pitt R.N., Taskin Alp Y., Shell I.A. (2021). The mental health consequences of work-life and life-work conflicts for STEM postdoctoral trainees. Front. Psychol..

[73] Al-Adawi S., Alameddine M., Al-Saadoon M., Al-Balushi A.A., Chan M.F., Bou-Karroum K., Al-Kindy H., Al-Harthi S.M. (2022). The magnitude and effect of work-life imbalance on cognition and affective range among the non-western population: A study from Muscat. PLoS ONE.

[74] van Lippe T.D., Lippényi Z. (2021). Investments in a Sustainable Workforce in Europe.

[75] Soares A.K., Goedert M.C., Vargas A.F. (2022). Mental health and social connectedness during the COVID-19 pandemic: An analysis of sports and E-sports players. Front. Psychol..

[76] Jung H.S., Jung Y.S., Yoon H.H. (2021). COVID-19: The effects of job insecurity on the job engagement and turnover intent of deluxe hotel employees and the moderating role of generational characteristics. Int. J. Hosp. Manag..

[77] Brough P., Timms C., Bauld R. Measuring work-life balance: Validation of a new measure across five Anglo and Asian samples. Proceedings of the 8th Australian Psychological Society Industrial & Organizational Conference (IOP).

[78] World Health Organization Wellbeing Measures in Primary Health Care: The DepCare Project: Report on a WHO Meeting. https://apps.who.int/iris/handle/10665/349766.

[79] Vargas-Jiménez E., Castro-Castañeda R., Tomás E.A., Centeno R.M. (2020). Job insecurity, family functionality and mental health: A comparative study between male and female hospitality workers. Behav. Sci..

[80] Grant C.A., Wallace L.M., Spurgeon P.C. (2013). An exploration of the psychological factors affecting remote E-worker’s job effectiveness, well-being and work-life balance. Empl. Relat..

[81] Yuen M. No Longer just the Breadwinner. https://www.thestar.com.my/news/focus/2021/06/20/no-longer-just-the-breadwinner.

[82] Gómez-Baya D., Lucia-Casademunt A., Salinas-Pérez J. (2018). Gender differences in psychological well-being and health problems among European health professionals: Analysis of psychological basic needs and job satisfaction. Int. J. Environ. Res. Public Health.

[83] Ministry of Human Resources Akta dan Polisi. https://www.mohr.gov.my/index.php/en/component/tags/tag/16-akta-dan-polisi.

[84] Kossek E.E. (2016). Implementing organizational work–life interventions: Toward a triple bottom line. Community Work Fam..

